# New Non-Destructive Surface Roughness Inspection of Additively Manufactured Metallic Parts Using Scanning Acoustic Microscopy

**DOI:** 10.3390/ma19214691

**Published:** 2026-11-04

**Authors:** Elham Alfuqara, Matthias Hien, Edward André Olivera Apaza, Stephan Brandl

**Affiliations:** Technology Campus Cham, Technische Hochschule Deggendorf, 93413 Cham, Germany; matthias.hien@th-deg.de (M.H.); edward.olivera-apaza@th-deg.de (E.A.O.A.); stephan.brandl@th-deg.de (S.B.)

**Keywords:** additive manufacturing, scanning acoustic microscopy (SAM), surface roughness, non-destructive testing, image processing

## Abstract

Although additive manufacturing enables the production of metallic components with complex geometries, accurately characterizing their surfaces remains challenging, especially in regions beyond the reach of conventional techniques. To address this challenge, this study investigates scanning acoustic microscopy (SAM) as an automated, non-destructive method for measuring the surface roughness of stainless steel 316L cubes fabricated by selective laser melting. A dedicated image-processing pipeline was developed to detect the water–specimen interface in SAM B-scan images using robust surface echo tracking and convert it into a calibrated surface profile. ISO-based spatial filtering was then applied to separate roughness from measurement noise, waviness, and surface form, enabling the calculation of different surface roughness parameters. To evaluate the proposed method, *R*_a_ was selected for validation using seven reference standards with nominal values ranging from 0.8 to 50μm. The influence of Gaussian filtering was also investigated by comparing two formulations. SAM successfully distinguished all seven roughness levels, with absolute deviations ranging from 0.327 to 1.772μm for the cutoff-matched formulation and from 0.199 to 4.075μm for the simplified formulation. Furthermore, repeatability measurements demonstrated mean coefficients of variation below 2% across all investigated specimens. Comparison between the proposed SAM pipeline, digital and laser confocal microscopes showed mean absolute deviations of 0.75, 1.30, and 2.85μm, respectively, from shared reference values. Building on this comparison, the optical instruments were used to assess SAM measurements on fabricated cubes without known reference values. Overall, the findings support SAM as a complementary method for quantitative surface roughness evaluation.

## 1. Introduction

Producing additively manufactured metal components that meet their functional requirements involves not only achieving the required geometry, but also obtaining a surface condition suitable for the intended application. Effectively controlling and monitoring this condition requires a clear understanding of how printing and post-processing parameters influence the final surface quality. Among the characteristics that define this quality, surface roughness is particularly important because it can directly affect dimensional accuracy, friction, fatigue behavior, subsequent processing, and the overall functional performance of additively manufactured components. Consequently, accurate and efficient surface roughness measurement is essential for evaluating and controlling the quality of these components.

Accurately measuring surface roughness is particularly challenging for metal additively manufactured components due to the complex nature of the surfaces produced during manufacturing. In their as-built condition, these surfaces are heterogeneous and direction-dependent, with features occurring across different spatial scales, including partially fused particles, weld-track ripples, balling, stair-stepping, deep valleys, and re-entrant structures. Consequently, a single profile measurement may not fully represent the overall surface condition [1,2]. Surface texture is commonly evaluated using profile parameters, such as *R*_a_, *R*_q_, and *R*_z_, or areal parameters, such as *S*_a_ and *S*_q_. ISO 21920-2 [3] defines the terms and parameters for profile measurements, while ISO 25178-2 [4] covers areal surface-texture measurements. ASTM F3624-23 [5] provides additional guidance specifically for measuring and characterizing the surface texture of metal powder-bed-fusion components.

Several established techniques are available for measuring these parameters, but each is suitable only under certain conditions. Stylus profilometry provides highly accurate and traceable measurements at a relatively moderate cost, but it requires direct contact with the surface and may affect delicate or loosely attached surface features [1,2]. Optical techniques, including coherence-scanning interferometry, confocal microscopy, focus-variation microscopy, structured-light measurement, and laser scanning, provide non-contact surface measurements and can capture areal topography. However, the required equipment can be expensive, and measuring large areas at a high resolution can require considerable time [1,2]. Moreover, measurements obtained using different instruments cannot always be compared directly because the results depend on the instrument resolution, measured area, surface orientation, treatment of missing data, and the filtering and form-removal procedures applied during processing [1].

Accessibility creates an additional challenge because stylus profilometry and conventional optical techniques cannot normally measure internal channels, lattice structures, or other non-line-of-sight surfaces. X-ray computed tomography (XCT) can characterize both external and inaccessible internal surfaces without damaging the component [6,7,8,9]. Nevertheless, XCT requires specialized and expensive equipment, as well as long measurement and reconstruction times. Its accuracy also depends on factors such as voxel size, focal-spot dimensions, surrounding material thickness, reconstruction settings, image noise, and the method used to identify the material boundary [6,7,8,9]. These practical and technical limitations created a need for an alternative method that could measure surface roughness quickly and non-destructively while providing acceptable accuracy at a reasonable cost. This need led us to investigate SAM as a potential surface roughness measurement method [10,11].

SAM provides a different non-destructive approach by using focused high-frequency acoustic waves to generate information about surface, subsurface, and interfacial features [10]. In additive manufacturing (AM) research, SAM has primarily been applied to porosity assessment and the detection of internal or interfacial discontinuities rather than to standardized surface roughness measurement [12,13,14]. Park et al., for example, used SAM as a reference technique for porosity evaluation and demonstrated that rough as-built surfaces reduce the signal-to-noise ratio of ultrasonic measurements [12,13], while subsequent investigations applied SAM to the inspection of porosity in directed-energy-deposited components using alternative coupling fluids [14]. Similarly, Yin et al. demonstrated that surface roughness significantly affects laser-ultrasonic propagation and defect-detection performance in LPBF-manufactured 316L stainless steel [15]. Although an earlier study showed that roughness information could be derived from slightly defocused acoustic microscopy images [16], it did not address additively manufactured surfaces or establish an ISO-compliant measurement procedure. More recently, SAM has been proposed as a potential quality-control technique for three-dimensional printing, but this work was conceptual rather than an experimental validation of parameters such as *R*_a_, *R*_q_, *S*_a_, or *S*_q_ [11]. Other acoustic approaches have also been investigated for estimating surface roughness. Feng et al. developed a model-based method that estimated surface roughness from acoustic emission signals recorded during pin-on-disc sliding tests. The method achieved an average error of approximately 14% compared with experimental roughness measurements under different operating conditions [17]. However, this method provides an indirect estimate based on signals generated during sliding contact and does not reconstruct a surface profile from SAM images. Therefore, previous research has not yet systematically validated SAM for quantitative roughness measurement of additively manufactured metallic surfaces. This research gap motivates the present study, in which surface profiles are extracted from SAM B-scan images and the resulting roughness parameters are validated using reference surfaces with known nominal values.

## 2. Materials and Methods

This study began with the fabrication of cubic specimens made of stainless steel 316L under standard printing conditions using a LASERTEC 30 SLM 3D printer (DMG MORI Additive GmbH, Bielefeld, Germany [18]). Some specimens were retained in their as-built condition, while selected specimens were electropolished using different voltages and polishing times, providing both untreated and post-processed surfaces for the investigation. Surface roughness reference specimens with known nominal values were also examined to support the quantitative validation of the method. The following sections describe the SAM measurement principle, image acquisition procedure, and each stage of the proposed processing pipeline.

### 2.1. Principles of SAM-Based Surface Profiling

SAM is a non-destructive imaging technique that uses focused high-frequency ultrasonic waves to examine the surface and internal structure of a material. A typical SAM system consists of a piezoelectric transducer, an acoustic lens, a mechanical scanning unit, and a signal-acquisition and image-processing system. The transducer generates short ultrasonic pulses and focuses them onto the specimen through a coupling medium, which is commonly water. When an acoustic wave reaches the specimen, part of its energy is reflected at the surface, while the remaining energy is transmitted into the material. Further reflections may occur at internal interfaces, pores, cracks, inclusions, or other discontinuities [10]. In the present study, the reflection from the water–specimen interface is particularly important because its position in the acquired signal represents the location of the specimen surface. Tracking this reflection across consecutive lateral positions therefore provides the information required to reconstruct the surface profile.

The strength of the reflected signal mainly depends on the difference in acoustic impedance between the coupling medium and the specimen. The acoustic impedance *Z* is defined as:

(1) Z=ρv

where ρ is the material density and *v* is the speed of sound in the material. The difference between the acoustic impedances of different materials produces a strong reflection at the specimen surface, making the surface boundary visible in the SAM signal.

For an acoustic wave reaching an interface at normal incidence, the pressure-amplitude reflection coefficient *r* is given by:

(2) $$r=\frac{Z_{2}-Z_{1}}{Z_{2}+Z_{1}}$$

where $Z_{1}$ and $Z_{2}$ are the acoustic impedances of the coupling medium and the specimen, respectively. The magnitude of *r* describes the relative amplitude of the reflected pressure wave, while its sign indicates whether a phase inversion occurs. The corresponding intensity reflection coefficient *R*, which represents the fraction of incident acoustic energy reflected at the interface, is:

(3) $$R={\left|r\right|}^{2}={\left|\frac{Z_{2}-Z_{1}}{Z_{2}+Z_{1}}\right|}^{2}$$

These coefficients explain why the specimen surface appears as a distinct echo in the acquired data. However, the amplitude of this echo may vary across a rough surface because local slopes and surface irregularities can change the direction and strength of the reflected wave. The proposed image-processing pipeline must therefore detect the position of the surface reliably despite local variations in echo amplitude.

In addition to its amplitude, the arrival time of the reflected echo provides information about the position of the surface along the depth direction. For an acoustic pulse propagating through a medium at a sound velocity *v*, the distance *d* to the reflecting interface is calculated as

(4) $$d=\frac{vt}{2}$$

where *t* is the measured round-trip time and the factor of two accounts for the forward and return paths of the acoustic wave [10]. For the surface reflection examined in this study, *v* corresponds to the speed of sound in the water used as the coupling medium. Differences in the echo arrival time between neighboring lateral positions indicate differences in surface height. The time-of-flight information can therefore be converted into a depth profile from which the surface roughness parameters are subsequently calculated.

SAM data can be represented using different scan modes, as illustrated in Figure 1. An A-scan records echo amplitude over time, while a B-scan combines consecutive A-scans to produce a cross-sectional image showing lateral position and depth. A C-scan provides a plan-view image of the specimen surface or internal layers. Additional modes, including P-, X-, D-, and Z-scans, offer alternative views of the specimen [10].

In this study, B-scan images were selected because they preserve the cross-sectional position of the water–specimen interface and therefore provide the information required to extract a surface profile. The suitability of this scan mode was first observed in a B-scan acquired along a path crossing the three reference surfaces, as shown in Figure 2. The appearance of the surface echo changed clearly between the reference regions, with the vertical variation and irregularity of the detected interface increasing from the 12.5μm surface to the 25μm and 50μm surfaces. Although this initial observation was qualitative, it indicated that the B-scan retained information related to differences in surface roughness and therefore motivated the development of the proposed method for extracting surface profiles and calculating quantitative roughness parameters.

### 2.2. Proposed Surface Roughness Analysis Pipeline

To determine surface roughness from the acoustic measurements, we developed a processing pipeline that converts the SAM data into calibrated surface profiles and quantitative roughness parameters. The B-scan images were acquired using a SAM 301 scanning acoustic microscope (PVA TePla Analytical Systems GmbH, Westhausen, Germany) and processed through five main stages, as illustrated in Figure 3.

#### 2.2.1. SAM Image Acquisition

The acoustic scans were performed with a PVA TePla SAM 301 microscope using WinSAM 8 software (version 8.23.4.0). For the acquisition of the B-scan images, the microscope was equipped with a 125MHz transducer having a nominal focal length of 9mm. Using this setup, we examined two groups of samples representing known and unknown surface roughness. The first group consisted of seven surface roughness standard specimens with nominal *R*_a_ values of 0.8, 1.6, 3.2, 6.3, 12.5, 25, and 50μm, covering all available roughness values on the MICROSURF 334 reference plate manufactured by SMT-Messzeuge. The second group consisted of three additively manufactured cubic specimens: one with an as-built surface and two electropolished specimens treated at 8V for 8 and 20min, respectively. Figure 4 shows the standards plate and the acquisition process for the standards and cubic specimens using the SAM microscope.

Before acquiring the B-scans, the acoustic focus was adjusted to obtain a clear and strong reflection from the specimen surface. As illustrated in Figure 5a, the ultrasonic beam becomes progressively narrower as it approaches the focal plane, where the acoustic energy is concentrated within the smallest area. When the specimen surface coincides with this plane, the reflected signal reaches its highest intensity and the best lateral resolution is obtained. If the surface is positioned either above or below the focal plane, the beam interacts with it over a larger area, resulting in a weaker and less sharply defined reflection. The corresponding relationship between the transducer position and the reflected signal intensity is shown in Figure 5b.

To bring the specimen surface close to the focal plane, its expected time-of-flight position was first estimated using the nominal focal length of the transducer and the speed of sound in water. By rearranging the pulse–echo relationship introduced in Equation (4), the expected time of flight of the surface reflection at the focal position was calculated as:

(5) $$t_{\mathrm{focus}}=\frac{2f}{c_{w}}$$

where *f* is the transducer focal length and $c_{w}$ is the speed of sound in water. At a water temperature of approximately 20 °C, a sound speed of approximately 1480m/s was used. For the transducer focal length of 9mm, the expected time of flight to the surface and back was approximately 12.16μs. The transducer was then moved along the vertical axis until the surface echo appeared near the expected time of flight, after which its position was adjusted more precisely by monitoring the sharpness and intensity of the reflected signal. The receiver gain was also adjusted so that the surface peak selected by the active data gate reached approximately 70–80% of the displayed full-scale amplitude. This produced a strong surface response without saturating or clipping the signal. The main WinSAM 8 settings and the percentage scale used during this adjustment are presented in Figure 6.

Once the acoustic focus had been established, a C-scan was acquired to obtain a plan-view image of the specimen surface and select a representative 10mm×10mm region of interest. The C-scan was also used to define the positions and repetitions of the parallel B-scans within this area. The main dataset included the seven reference specimens and the three cubic specimens. For each specimen, 500 parallel B-scans were acquired across the selected area, and the complete acquisition was repeated once, resulting in 1000 B-scan images per sample and 10,000 images across the ten samples. Further details on the spatial arrangement and processing of these B-scans are provided in the pipeline step entitled Spatial Sampling of the Surface. Another dataset, hereafter referred to as the repeatability dataset, was collected to assess the precision and repeatability of the SAM method. For each of the seven reference specimens and three cubic specimens, 10 fixed measurement lines were selected, and 21 repeated B-scans per line were evaluated under unchanged measurement conditions. This resulted in 210 evaluated measurements per specimen and 2100 measurements across the complete repeatability study. Repeatedly measuring the same fixed lines reduced the influence of spatial surface variation and allowed the variation associated with the measurement procedure to be evaluated.

During B-scan acquisition, the surface-trigger function in WinSAM 8 was disabled. This function normally compensates for changes in the position of the upper specimen surface caused by tilt, bow, warping, dents, or similar geometrical variations by aligning the detected surface echoes within the acquisition window. Although this correction is useful when examining subsurface features, it is unsuitable for the present application because variations in the surface position contain the information required for roughness evaluation. Preliminary measurements showed that activating the surface trigger produced an artificially flattened representation of the surface and removed some of its relevant height variations. The surface trigger was therefore disabled for all measurements so that the original surface geometry was preserved in the B-scan images and could subsequently be extracted and analyzed.

#### 2.2.2. Surface Profile Extraction

The surface profile extraction step aimed to identify the water–stainless steel interface in each SAM B-scan and determine its axial position along the lateral scanning direction. Let I(x,y) denote the grayscale image intensity at lateral pixel position *x* and axial pixel position *y*, where *x* represents the horizontal scanning direction and *y* represents the depth direction, increasing downward in the B-scan image.

The water–stainless steel interface produces a reflected ultrasound signal that appears as an intensity transition in the B-scan. However, the image may also contain additional echoes arising from subsequent acoustic reflections. Furthermore, the intensity and position of the surface echo can vary considerably, particularly for rough specimens, due to local surface orientation, acoustic scattering, and variations in the reflected signal. Consequently, the strongest reflection does not necessarily represent the actual specimen surface.

To address this challenge, a multi-candidate surface tracking approach was developed. Rather than selecting a single reflection independently in each image column, the algorithm first identified several possible echo positions and then evaluated their spatial consistency across neighboring columns. The procedure consisted of image preparation, identification of potential echo onsets, continuous surface tracking, and refinement and quality assessment of the extracted profile.

##### Step 1: Image Preparation and Identification of the Echo Region

The first step was to identify the part of the B-scan containing the relevant acoustic reflections. This reduced the influence of background noise and restricted the subsequent analysis to the region in which the specimen surface was expected.

For this purpose, an upper axial search region was defined according to the B-scan acquisition configuration and the expected position of the surface echoes. The maximum search depth, denoted by $N_{\mathrm{top}}$, was selected to include the relevant reflections within the available image height. This allowed the procedure to accommodate the different axial ranges used during image acquisition.

Before identifying the echoes, the selected image region was smoothed using a 3×3 Gaussian kernel, with standard deviations of approximately 0.65 pixels in the lateral and axial directions. This preprocessing reduced isolated intensity fluctuations that could otherwise be mistaken for acoustic responses. The resulting image is denoted by $I_{G}\left(x,y\right)$.

Since the B-scan may contain a weak background signal even in regions without visible reflections, the background intensity was estimated before detecting the echoes. The median intensity within the upper fifth of the search region was used as the background level, $I_{\mathrm{bg}}$. The corresponding background variability was estimated using the median absolute deviation (MAD):

(6) $$\sigma_{\mathrm{bg}}=\max\left(1,\;1.4826\;\mathrm{median}\left|I_{G}\left(x,y\right)-I_{\mathrm{bg}}\right|\right)$$

where the median is evaluated over the selected background region. This provided a noise estimate that was less sensitive to occasional bright pixels than a calculation based on the maximum intensity. The background level was then subtracted from the smoothed image to obtain the non-negative signal:

(7) $$A\left(x,y\right)=\max\left[I_{G}\left(x,y\right)-I_{\mathrm{bg}},\;0\right]$$

The resulting signal A(x,y) represents the intensity above the estimated background level and was used for the subsequent echo detection.

Next, the algorithm determined the axial region containing significant acoustic responses. For each image row, the 98th percentile of the background-corrected intensity was calculated across the lateral direction. This provided a measure of the signal strength at each depth without relying exclusively on the brightest pixel.

A row was considered active when this value exceeded the largest of three thresholds: an absolute intensity threshold of 6, 10% of the maximum row-wise percentile, or five times the estimated background variability $\sigma_{\mathrm{bg}}$.

The axial search interval was then defined from 25 pixels above the first active row to 15 pixels below the last active row, subject to the limits of the initial search region. In this way, the search interval included the main acoustic responses while allowing their axial positions to vary across the B-scan. This was particularly important for inclined surfaces and specimens with pronounced local height variations.

##### Step 2: Identification of Potential Surface Echoes

Once the relevant echo region had been identified, the algorithm examined each lateral image column to locate possible surface reflections.

A B-scan column may contain several acoustic responses at different depths. Although the first surface reflection is expected to occur earlier than subsequent echoes, it is not necessarily the strongest. Therefore, selecting the brightest response could lead to incorrect surface positions, particularly when the intensity of the first echo decreases.

Instead, the algorithm identified several possible echo positions in each column. These positions, referred to as *echo candidates*, were determined by comparing the background-corrected intensity A(x,y) with a noise-referenced threshold:

(8) $$T_{e}=\max\left(4,\;4.5\sigma_{\mathrm{bg}}\right)$$

The threshold was based on the estimated background noise rather than the maximum intensity within each column. This allowed relatively weak first echoes to remain detectable even when stronger reflections appeared at greater depths.

To avoid identifying isolated bright pixels as valid reflections, an echo candidate was accepted only when at least three consecutive axial samples exceeded $T_{e}$. The first pixel of this sequence was identified as the *echo onset*, representing the beginning of the detected acoustic response.

Candidates separated by fewer than five axial pixels were grouped to avoid interpreting small interruptions within the same reflection as separate echoes.

The remaining candidates were ordered according to their axial positions, beginning with the earliest detected response. Up to seven of the earliest candidates were retained for each column. Each candidate *j* in column *x* was characterized by its axial position $p_{x,j}$ and its local echo strength $e_{x,j}$, defined as the maximum background-corrected intensity within the seven axial samples following its onset.

At this stage, the algorithm had identified several possible surface positions in each column. However, the candidates could not yet be considered the final surface profile, since some of them could correspond to subsequent acoustic reflections.

##### Step 3: Continuous Surface Tracking

The next step was to determine which of the detected candidates most likely belonged to the physical surface.

Rather than selecting the earliest or strongest candidate independently in every column, the algorithm considered how the candidate positions changed across neighboring columns. This was necessary because the intensity of the first echo could vary along the surface, and an isolated incorrect selection could introduce an artificial peak or valley into the extracted profile.

The surface was therefore identified using dynamic programming, which evaluates possible connections between candidates in consecutive image columns and selects the trajectory with the lowest overall cost.

The selection was based on three considerations. First, earlier echoes were preferred because the physical surface corresponds to the first acoustic reflection. Second, candidates with stronger local acoustic evidence were favored over weaker responses. Third, changes in axial position and unnecessary switching between candidate indices were penalized to discourage transitions between different echoes.

For candidate *j* in column *x*, the cumulative cost was defined as:

(9) $$C_{x}\left(j\right)=L_{x}\left(j\right)+\min_{k}\left[C_{x-1}\left(k\right)+M\left(|p_{x,j}-p_{x-1,k}|\right)+S\left(j,k\right)\right]$$

where $C_{x}\left(j\right)$ represents the minimum cumulative cost of reaching candidate *j* in the current column, and *k* denotes a candidate in the preceding column.

The local cost $L_{x}\left(j\right)$ describes the preference for earlier and sufficiently strong echoes. The term *M* penalizes changes in axial position between neighboring columns, while S(j,k) discourages unnecessary switching between candidate indices. In the implemented algorithm, these terms were expressed as:

(10) $$L_{x}\left(j\right)=2.8j+1.8\left(1-\frac{e_{x,j}}{\max_{k}e_{x,k}}\right)$$

(11) M(δ)=0.35min(δ,25)+0.20max(δ−25,0)

(12) $$S(j,k)=1.8\;1_{j\neq k}$$

where the candidate indices begin at j=0, δ represents the absolute axial difference between two consecutive candidate positions in pixels, and $1_{j\neq k}$ equals one when the candidate indices differ and zero otherwise.

The local candidate cost $L_{x}\left(j\right)$, defined in Equation (10), favors earlier echo candidates by assigning them lower costs while also considering their relative signal strength. The axial depth-change penalty M(δ), given in Equation (11), discourages abrupt changes in surface depth between neighboring columns that could indicate incorrect transitions between echoes. However, for depth differences greater than 25 pixels, the penalty increases more slowly, allowing the algorithm to accommodate pronounced local surface variations. Finally, the candidate-switching penalty S(j,k), defined in Equation (12), discourages unnecessary changes between candidate indices in consecutive columns, helping the algorithm maintain a consistent surface trajectory.

After the cumulative costs had been calculated, the minimum-cost trajectory was recovered by tracing the selected candidate connections backward through the image columns.

Importantly, this procedure did not assume that the specimen surface was straight or followed a predefined mathematical shape. Instead, the detected surface was determined from the acoustic responses in each column, while the continuity constraints reduced the likelihood of incorrect transitions between reflections. This allowed the method to follow both inclined surfaces and local surface irregularities.

##### Step 4: Surface Refinement and Quality Control

After identifying the surface trajectory, the detected echo positions were refined using linear interpolation between neighboring axial samples at the threshold crossing. This allowed the surface position to be estimated with subpixel precision. Moreover, no additional Gaussian smoothing was applied to the extracted profile to preserve genuine surface irregularities, particularly for specimens with higher roughness. Noise reduction and scale separation were instead addressed during the subsequent profile-filtering stage.

Although the tracking algorithm was designed to follow the first surface echo, some regions could remain uncertain due to weak reflections, acoustic scattering, or local surface orientation. When no valid echo candidate was detected, the missing position was interpolated to maintain a continuous profile for further processing. However, these interpolated positions were not considered directly measured surface coordinates.

To assess the reliability of the extracted profile, the algorithm flagged columns where a later echo candidate was selected, or where the depth difference between neighboring columns exceeded 14 pixels, or where the selected position differed from the earliest available candidate by more than 8 pixels. A B-scan was marked for visual inspection if more than 2% of its columns were flagged or if any echo positions were missing. These checks helped identify potentially incorrect surface detections but were not intended to quantify measurement uncertainty.

##### Step 5: The Extracted Surface Profile

The final output of the tracking procedure was a sequence of surface-depth coordinates corresponding to the lateral positions of the B-scan. The extracted profile was represented in pixel coordinates as:

(13) $$P_{\mathrm{px}}=\left\{\left(x,p(x)\right)\;|\;x=0,1,\ldots ,W-1\right\}$$

where *W* is the image width and p(x) represents the selected axial position of the surface at lateral position *x*. Where possible, the profile included subpixel coordinates obtained from interpolation of the detected echo onset.

For visual verification, the extracted trajectory was overlaid on the original grayscale B-scan. Regions identified by the quality control procedure were highlighted to facilitate inspection of potentially incorrect echo assignments or missing surface reflections.

Figure 7 illustrates the extraction procedure using a representative B-scan, showing the original image, the detected surface boundary, and the resulting depth profile. The conversion of these pixel coordinates into physical dimensions is described in the following subsection.

#### 2.2.3. Spatial Calibration and ISO-Based Profile Filtering

Following surface profile extraction, the detected surface trajectory was converted from image pixels into a profile expressed in physical units (mm). The calibrated profile was then subjected to scale separation and residual-form removal before calculation of the roughness parameters. These operations were applied after echo tracking and were not used to determine the position of the water–specimen interface.

For lateral calibration, the sampling interval was defined in the WinSAM 8 acquisition software. For the measurements considered here, it was set to Δx=2.0μm/pixel. The lateral coordinate of image column *i* was therefore calculated as:

(14) $$x_{i}=i\Delta x$$

For depth calibration, the axial sampling interval was obtained from the acquisition time gate and the speed of sound in water. For a pulse–echo measurement, the recorded propagation time corresponds to the outward and return paths, giving an axial increment of $\Delta z=c_{w}\Delta t/2$. The B-scans typically comprised $N_{y}=1000$ axial samples over a time gate of T=1000ns. For the 50μm reference standard, a longer time gate of 2000ns with 2000 axial samples was used. Both configurations provided the same temporal sampling interval:

(15) $$\Delta t=\frac{T}{N_{y}}=1.0\;\mathrm{ns}/\mathrm{sample}$$

Using $c_{w}\approx 1480\;m/s$ for water at approximately 20 °C, the corresponding axial sampling interval was

(16) $$\Delta z=\frac{c_{w}\Delta t}{2}=0.74\;\mu m/\mathrm{pixel}$$

Thus, the relative depth coordinate for the detected surface sample at column *i* was

(17) $$z_{i}=p\left(i\right)\Delta z.$$

The constant offset associated with the acquisition gate start was omitted, as the roughness evaluation depends only on relative height variations.

The evaluation length was selected based on the lateral image dimension. For each B-scans used in this study, the lateral image dimension was 5000 pixels. At 2.0μm/pixel, this corresponds to a nominal scan width of 10mm. The algorithm selected a central window of 10mm from the calibrated profile, which therefore retained the complete available lateral profile for these images.

Profile filtering was then applied to the calibrated surface profile. The calibrated surface profile contains variations over different spatial scales, including short-wavelength fluctuations, surface roughness, longer-wavelength waviness, and residual form. To separate these contributions, the extracted profile was processed using an S-filter followed by an L-filter, after which the remaining linear form was removed. The S-filter suppresses the shortest lateral wavelengths, whereas the L-filter separates the longer-wavelength component from the roughness profile.

The selection of the filter nesting indices depends on the specified upper tolerance limit *U* of the roughness parameter. For the *R*_a_ parameter, ISO 21920-3 [19] defines the default settings listed in Table 1. The profile L-filter nesting index $N_{\mathrm{ic}}$ is conventionally associated with the cutoff $\lambda_{c}$, whereas the profile S-filter nesting index $N_{\mathrm{is}}$ is associated with $\lambda_{s}$.

Thus, the appropriate nesting must be selected according to the applicable specification rather than held at a common value for all reference specimens. The table includes the three ranges relevant to the specimens used in this study; ISO 21920-3 [19] also defines additional setting classes for smaller *U* values. For the numerical profile processing, two approaches were considered for determining the Gaussian filter width. The initial implementation, hereafter referred to as the *simplified Gaussian formulation*, used a simplified relationship between the selected nesting index λ and the lateral sampling interval Δx:

(18) $$\sigma_{\mathrm{px}}^{\mathrm{simp}}=\frac{\lambda }{2\Delta x}$$

To evaluate the influence of this approximation on the calculated surface roughness, a second approach, hereafter referred to as the *cutoff-matched Gaussian formulation*, was investigated using the Gaussian cutoff relationship defined in ISO 16610-21 [20]:

(19) $$\sigma_{\mathrm{px}}^{\mathrm{ISO}}=\frac{\sqrt{2\ln2}}{2\pi }\frac{\lambda }{\Delta x}$$

Here, $\sigma_{\mathrm{px}}$ denotes the Gaussian standard deviation in pixels, λ is the selected filter nesting index, and Δx is the lateral sampling interval. Both approaches were applied to the same extracted and calibrated surface profiles, using identical nesting indices and subsequent processing steps. This comparison was conducted to quantify the influence of the Gaussian filter width on the resulting roughness parameters and assess its implications for comparison with conventional surface roughness measurements.

For each approach, the S-filter was first applied to the calibrated height profile z[i]:

(20) $$z_{S}\left[i\right]=G_{\sigma_{s}}\left\{z\left[i\right]\right\}$$

where $G_{\sigma }$ represents one-dimensional Gaussian low-pass filtering. The L-filter was subsequently applied to estimate the longer-wavelength component, which was subtracted from the S-filtered profile:

(21) $$\begin{array}{cc} z_{L}\left[i\right] & =G_{\sigma_{l}}\left\{z_{S}\left[i\right]\right\},\\ z_{R}\left[i\right] & =z_{S}\left[i\right]-z_{L}\left[i\right] \end{array}$$

The resulting roughness profile $z_{R}\left[i\right]$ was then used for the subsequent residual-form removal operation. By applying both Gaussian-width formulations to identical profiles, the effect of the filtering approach could be evaluated independently of differences in surface detection and spatial calibration.

Following the profile-filtering stage, the remaining form was removed using the F-operation. After S- and L-filtering, a residual linear component could remain in the roughness-band profile. The implemented F-operation estimated this component using least-squares regression:

(22) $$z_{\mathrm{trend}}\left(x_{i}\right)=ax_{i}+b$$

and subtracted it to obtain the final profile for roughness evaluation:

(23) $$z_{r}\left[i\right]=z_{R}\left[i\right]-z_{\mathrm{trend}}\left(x_{i}\right)$$

The linear fit was applied only after surface detection and scale separation, as both the reference standards and the additively manufactured cubic specimens have nominally planar surfaces. This operation removes any residual linear tilt without assuming that the local surface height variations are linear. It must therefore be distinguished from the first-echo tracking procedure, which did not impose a linear surface model. Figure 8 shows the calibrated surface profile, the S- and L-filtered components, and the final roughness profile following residual-form removal. Roughness parameters were subsequently calculated from $z_{r}\left[i\right]$ as described in the following subsection.

#### 2.2.4. Roughness Parameter Calculation

Surface roughness parameters provide numerical descriptions of the height variations contained in the final roughness profile. As illustrated in Figure 9, *R*_a_ represents the average magnitude of the profile deviations from the mean line, while *R*_q_ gives greater weight to large deviations. The parameters *R*_p_ and *R*_v_ describe the highest peak and deepest valley, respectively, whereas *R*_t_ represents the total vertical distance between them. Together, these parameters provide complementary information about the magnitude and distribution of surface irregularities [3].

In the implemented pipeline, these parameters were calculated from the final roughness profile $z_{r}\left[i\right]$, which was centred approximately on the mean line after removal of the fitted linear form. For a profile containing *N* uniformly spaced points, the parameters were calculated as:

(24) $$R_{a}=\frac{1}{N}\sum_{i=1}^{N}\left|z_{r}\left[i\right]\right|$$

(25) $$R_{q}=\sqrt{\frac{1}{N}\sum_{i=1}^{N}z_{r}^{2}\left[i\right]}$$

(26) $$R_{p}=\max_{1\leq i\leq N}\left(z_{r}\left[i\right]\right)$$

(27) $$R_{v}=-\min_{1\leq i\leq N}\left(z_{r}\left[i\right]\right)$$

(28) $$R_{t}=R_{p}+R_{v}$$

The algorithm also returned a quantity denoted as *R*_z_ (approx.), calculated as *R*_p_ + *R*_v_. Because this expression is identical to *R*_t_, it does not represent the standardized *R*_z_, which requires evaluation over defined sampling lengths. It was therefore treated only as an approximate peak-to-valley value and not as an ISO-compliant *R*_z_ result. All parameters were calculated in micrometres from the complete 10mm profile and were subsequently used for spatial mapping and validation.

#### 2.2.5. Spatial Mapping

The previous processing steps were applied to a single B-scan image, representing one measurement line across the surface of a reference standard or cubic specimen. However, the roughness obtained from one line may not adequately represent the complete surface because surface characteristics can vary between different positions. This is particularly relevant for the reference standards investigated in this study, whose C-scan images, shown in Figure 10, reveal spatial variations in the acoustic response across their surfaces. Consequently, individual measurement lines may not capture the full variability of the surface. Multiple parallel B-scans were therefore evaluated to characterize these spatial variations and obtain a more representative assessment of the measured area.

For each reference standard and cubic specimen, a nominal surface area of 10mm×10mm was examined. The spatial sampling strategy is illustrated in Figure 11. Figure 11a shows the SAM C-scan of the complete measurement area, while Figure 11b highlights one of the B-scan measurement lines. To evaluate the complete area, 500 parallel line positions were sampled at a constant spatial offset of 0.02mm. Each line was scanned twice under the same measurement conditions, resulting in 1000 B-scan images for each evaluated area. The distribution of the 500 line positions across the surface is shown in Figure 11c.

Before processing, the B-scan images were sorted according to the line and B-scan numbers contained in their filenames. This sorting preserved the spatial order in which the images were acquired. The complete processing pipeline, including surface profile extraction, spatial calibration, S- and L-filtering, form removal, and roughness parameter calculation, was then applied independently to every B-scan.

For each measurement line, the roughness parameters *R*_a_, *R*_q_, *R*_p_, *R*_v_, and *R*_t_ were calculated from the corresponding final roughness profile. Each calculated value was assigned to the spatial position of its B-scan, producing a one-dimensional spatial distribution of every roughness parameter across the evaluated area.

The final result for each evaluated area was obtained by first averaging the two repeated B-scan measurements at each physical line position, followed by calculating the arithmetic mean across all line positions. For a roughness parameter *P*, where *P* represents *R*_a_, *R*_q_, *R*_p_, *R*_v_, or *R*_t_, the mean at each line position was calculated as

(29) $$P_{j}=\frac{P_{j,1}+P_{j,2}}{2}$$

where $P_{j,1}$ and $P_{j,2}$ are the parameter values obtained from the two repeated B-scans at the *j*-th line position. The area-level mean was then calculated as

(30) $$\bar{P}=\frac{1}{M}\sum_{j=1}^{M}P_{j}$$

where $P_{j}$ is the mean parameter value at the *j*-th physical line position and M=500 is the total number of evaluated line positions.

The spatial variation of each parameter across the measured area was described using the sample standard deviation of the paired line means:

(31) $$s_{P}=\sqrt{\frac{1}{M-1}\sum_{j=1}^{M}{\left(P_{j}-\bar{P}\right)}^{2}}$$

The mean value provided a representative roughness result for the complete evaluated area, while the standard deviation described the variation among the physical measurement lines.

All line level results, their corresponding spatial positions, and the area-level summary statistics were exported to CSV files. These stored results were subsequently used for the validation of the proposed SAM-based surface roughness measurement method.

#### 2.2.6. Validation and Measurement Performance Assessment

Evaluating a measurement system requires consideration of different aspects of measurement quality. In this study, two complementary aspects were considered: accuracy and repeatability. Although they are related, they describe different properties of a measurement method and were therefore evaluated separately:

- Accuracy and agreement with a reference value: accuracy describes the overall closeness of a measurement to an accepted reference value. A complete assessment of accuracy requires a traceably calibrated reference value with a stated uncertainty, together with consideration of the measurement uncertainty. Agreement, in contrast, describes the observed difference between a measured value and an available reference or nominal value. It can be quantified directly using signed, absolute, and relative deviations. Close agreement provides an indication of accuracy, but it does not constitute a complete determination of accuracy when the uncertainty of the reference value is unknown [21].
- Repeatability and measurement precision: repeatability describes how closely repeated measurements agree when they are performed under unchanged conditions. It is an aspect of measurement precision and can be quantified using measures of dispersion, such as the standard deviation and coefficient of variation. A low coefficient of variation indicates that repeated results are closely grouped, but it does not indicate whether they agree with a reference value.

Based on this distinction, the measurement performance assessment for the proposed pipeline comprised three complementary components:

1. Reference-based evaluation: The reference specimens were used to examine the agreement of the three measurement methods with known nominal *R*_a_ values. This evaluation was necessary to determine how closely the SAM, digital-microscope, and laser-confocal measurements approached the expected roughness levels. Agreement was quantified using signed, absolute, and relative deviations. Because the uncertainty associated with the nominal values was unavailable, this evaluation provided an indication of accuracy rather than a complete metrological determination [21].
2. Inter-instrument comparison of the additively manufactured cubes: The cubes were measured using SAM and the two optical instruments to compare their performance on additively manufactured surfaces. The VHX-7000 digital microscope and VK-X3050 laser confocal microscope (KEYENCE CORPORATION, Osaka, Japan) were selected because optical systems are established tools for surface roughness measurement. Before the cube measurements, the two optical instruments and SAM were evaluated using the same reference specimens. This established how closely each method reproduced the nominal *R*_a_ values and provided a basis for interpreting the subsequent comparison on the cubes. To improve comparability, all measurements were performed on the same cube side using matched evaluation-area dimensions, evaluation lengths, and profile filtering settings. However, because the cubes had no independently established reference values, the optical measurements could not provide an absolute validation of SAM. The comparison was therefore used to identify systematic differences among the methods and to determine whether SAM produced results consistent with the established optical systems.
3. Repeatability assessment: Repeated SAM measurements were performed at fixed surface positions under unchanged conditions to evaluate the consistency of the measurement procedure. This assessment was necessary to separate short-term measurement variability from differences caused by the selected surface position or disagreement with a reference value. Repeatability was quantified using the standard deviation and coefficient of variation. It was evaluated independently of reference agreement because closely grouped repeated measurements do not necessarily indicate that the measured value is close to the reference value [21].

Together, these assessments examined agreement with nominal reference values, comparability with established measurement systems, and short-term measurement precision.

The reference-based evaluation was conducted using seven surface roughness reference specimens with nominal *R*_a_ values of 0.8, 1.6, 3.2, 6.3, 12.5, 25, and 50μm. All seven specimens were measured using the proposed SAM method, while the specimens with nominal values of 12.5, 25, and 50μm were additionally evaluated using a Keyence VK-X3050 laser confocal microscope and a Keyence VHX-7000 digital microscope. The evaluation focused on *R*_a_ as the common parameter for comparing the measurement results with the nominal reference values.

The nominal values provided a common basis for evaluating measurement deviations across the investigated roughness range. However, because the nominal values were not accompanied by calibration uncertainties, the comparison was interpreted as an indication of accuracy rather than a complete metrological determination of accuracy.

For each measurement method *d* and reference specimen *k*, the signed deviation between the mean measured value and the nominal value was calculated as:

(32) $$b_{d,k}={\bar{R}}_{a,d,k}-R_{a,\mathrm{nom},k}$$

where ${\bar{R}}_{a,d,k}$ is the mean measured value and $R_{a,\mathrm{nom},k}$ is the nominal roughness of the reference specimen. Positive values indicate an overestimation of the nominal value, whereas negative values indicate an underestimation. The magnitude of the deviation was expressed using the absolute deviation

(33) $$E_{\mathrm{abs},d,k}=\left|{\bar{R}}_{a,d,k}-R_{a,\mathrm{nom},k}\right|$$

and the relative deviation:

(34) $$E_{\mathrm{rel},d,k}=\frac{\left|{\bar{R}}_{a,d,k}-R_{a,\mathrm{nom},k}\right|}{R_{a,\mathrm{nom},k}}\times 100\%$$

The absolute deviation expresses the difference in micrometres, whereas the relative deviation expresses this difference as a percentage of the nominal roughness value.

For the inter-instrument comparison of the additively manufactured cubes, the additively manufactured cubes had no independently established reference *R*_a_ values and could therefore not be used to determine the accuracy of the proposed method directly. Instead, the cubes were measured using SAM, the VK-X3050 laser confocal microscope, and the VHX-7000 digital microscope, and the resulting *R*_a_ values were compared among the three instruments.

This inter-instrument comparison was used to identify systematic agreement or differences among the measurement methods when applied to additively manufactured surfaces. Differences between the instruments were not interpreted as measurement errors because no accepted reference values were available to determine which result was closest to the actual roughness. The cube measurements therefore demonstrate the practical application of the proposed method and its relationship to the two established optical measurement systems, rather than providing an independent validation of its accuracy.

Repeatability was assessed using repeated measurements performed at fixed surface positions under unchanged measurement conditions. The reference-specimen repeatability dataset described previously was used for this purpose, and the same statistical procedure was applied to the repeated measurements of the additively manufactured cubes. Evaluating fixed positions reduced the influence of spatial variations in surface roughness and allowed the variability associated with the measurement procedure itself to be examined.

For specimen *k* and measurement line *l*, the mean *R*_a_ value obtained from the repeated measurements was calculated as:

(35) $${\bar{R}}_{a,k,l}=\frac{1}{n_{k,l}}\sum_{r=1}^{n_{k,l}}R_{a,k,l,r}$$

where $n_{k,l}$ is the number of repeated measurements and $R_{a,k,l,r}$ is the result of repetition *r*. The sample standard deviation was calculated as:

(36) $$s_{k,l}=\sqrt{\frac{1}{n_{k,l}-1}\sum_{r=1}^{n_{k,l}}{\left(R_{a,k,l,r}-{\bar{R}}_{a,k,l}\right)}^{2}}$$

and the coefficient of variation was calculated as:

(37) $$CV_{k,l}=\frac{s_{k,l}}{{\bar{R}}_{a,k,l}}\times 100\%$$

The coefficient of variation expresses the standard deviation relative to the mean roughness value and therefore enables repeatability to be compared across specimens with different roughness levels. A lower CV indicates lower relative variation and better short-term repeatability under the investigated conditions. However, it does not indicate agreement with the nominal value. Consequently, a low coefficient of variation can coexist with a larger relative deviation: the former reflects consistency among repeated measurements, whereas the latter reflects agreement with the reference value. The outcomes of the reference-based evaluation, inter-instrument comparison, and repeatability assessment are presented separately in Section 3.

## 3. Results

Following the evaluation framework described in the previous section, the results are presented in three complementary parts. First, the *R*_a_ values obtained using SAM are compared with the nominal values of seven surface roughness reference standards, covering a range from 0.8 to 50μm. The influence of the simplified and cutoff-matched Gaussian-filter formulations on the calculated roughness values is also evaluated. For the 12.5, 25, and 50μm reference standards, additional measurements obtained using the Keyence VHX-7000 digital microscope and the Keyence VK-X3050 laser confocal microscope are included. Deviations from the nominal values are used to assess the measurement performance across the investigated roughness range.

Second, the measurements obtained from the additively manufactured cubes are compared across the three instruments. Because the cubes had no independently established reference values, this comparison is used to examine inter-instrument agreement and method-dependent differences rather than to determine measurement accuracy.

Finally, the repeatability of the SAM method is evaluated for both the reference specimens and the additively manufactured cubes using repeated measurements at fixed surface positions. The standard deviation and coefficient of variation are used to describe the consistency of the SAM measurements independently of their agreement with a reference value.

### 3.1. Reference-Based Evaluation

This evaluation was conducted using seven surface roughness standards with nominal *R*_a_ values of 0.8, 1.6, 3.2, 6.3, 12.5, 25, and 50μm. This wider range was investigated to assess the response of the proposed SAM method at different roughness levels and to identify variations in measurement deviation.

For each standard, the extracted SAM surface profiles were processed using both the simplified and cutoff-matched Gaussian-filter formulations described in the previous sections. This comparison was performed to assess the influence of Gaussian filter width on the calculated *R*_a_ values. Measurements obtained using the VHX-7000 and VK-X3050 were also included for the 12.5, 25, and 50μm standards. Since optical measurements were not available for the remaining four standards, the extended evaluation was based on the comparison between the SAM results and the nominal roughness values, while direct inter-instrument comparison was restricted to the three common reference standards.

The measured *R*_a_ values and their absolute deviations from the corresponding nominal values are summarized in Table 2.

The SAM measurements increased with the nominal roughness values under both Gaussian-filter formulations, demonstrating that the proposed method could distinguish all seven investigated roughness levels. However, the deviations from the nominal values varied across the investigated range. To examine this behavior, the signed deviation was calculated as:

(38) $$\Delta R_{a}=R_{a,\mathrm{measured}}-R_{a,\mathrm{nominal}}$$

where negative values indicate underestimation and positive values indicate overestimation. Figure 12 presents the deviations obtained using both Gaussian-filter formulations.

The measurement deviations varied across the seven reference standards without following a consistent trend with changing roughness. With the cutoff-matched Gaussian formulation, the largest underestimation occurred at 6.3μm, with a deviation of −1.772μm, whereas the 12.5 and 25μm standards showed smaller deviations. At 50μm, both filtering approaches overestimated the nominal value, particularly the simplified formulation, which produced a deviation of +4.075μm. These differences may be related to variations in the surface characteristics of the reference specimens. In particular, fine surface features may be insufficiently represented by the fixed lateral sampling interval, while the more pronounced surface irregularities of rougher specimens may introduce greater variability in acoustic echo localization. Together, these effects may influence the extracted profiles differently across the investigated roughness range.

The comparison between the two Gaussian formulations further shows that the filtering procedure influences the measured *R*_a_. The cutoff-matched formulation consistently produced lower values because the two approaches use different Gaussian widths, affecting the surface wavelengths retained after S- and L-filtering. The magnitude of this effect also depends on the selected nesting indices, which increase with the investigated roughness range. Although the cutoff-matched formulation consistently produced lower *R*_a_ values, this did not result in smaller deviations for every reference standard. The influence of Gaussian filtering on measurement deviation therefore varied across the investigated surfaces.

In addition to the area-averaged results, the spatial distribution of *R*_a_ was examined to investigate variations between individual measurement lines. Figure 13 presents representative results for the 1.6, 6.3, and 25μm reference standards. These specimens were selected to illustrate spatial variations at different roughness levels. Each plotted value represents the mean of two repeated B-scans acquired at the corresponding physical line position. The observed spatial variations also support the nonuniform surface response previously illustrated by the C-scan images in Figure 10.

The three reference standards with nominal *R*_a_ values of 12.5, 25, and 50μm were evaluated using all three instruments. Across these standards, the mean absolute deviation from the respective nominal values was 0.75μm for SAM using the cutoff-matched Gaussian formulation, compared with 1.30μm for the VHX-7000 and 2.85μm for the VK-X3050. These results demonstrate that the proposed SAM pipeline achieved a comparable level of measurement performance to established optical surface roughness measurement techniques, supporting its potential for quantitative roughness assessment. The comparison also provides a basis for using the two optical instruments to assess inter-instrument agreement on the additively manufactured cubic specimens, for which no independent reference roughness values were available.

Overall, the results demonstrate the potential of the proposed SAM pipeline for quantitative surface roughness measurement across a wide range of roughness levels, with performance comparable to established optical measurement techniques. The findings also highlight the importance of appropriate profile filtering for reliable roughness evaluation.

### 3.2. Evaluation of the Additively Manufactured Cubes

Following the reference-based evaluation, the proposed SAM method was applied to three additively manufactured cubes. Since the actual surface roughness of these specimens was unknown, their measurement accuracy could not be evaluated directly. Therefore, the VHX-7000 digital microscope and VK-X3050 laser confocal microscope, which had also been evaluated using the reference standards, were used to provide independent measurements for comparison with SAM. This allowed the consistency of the SAM results with established optical techniques to be assessed. The resulting *R*_a_ values are presented in Table 3.

The results show that SAM produced *R*_a_ values of comparable magnitude to those measured by the two optical instruments across the three cubes. The mean *R*_a_ across the specimens was 12.05μm for SAM, compared with 11.46μm for the VHX-7000 and 14.99μm for the VK-X3050. SAM therefore showed closer agreement with the digital microscope, with a mean absolute difference of 0.74μm, compared with 2.94μm for the laser confocal microscope. Notably, all three methods measured higher roughness on Cube 3 than on Cube 1, indicating a consistent overall trend despite differences in the measured values.

The remaining differences between the instruments may arise from their different measurement principles, spatial resolutions, evaluated surface regions, and profile-processing procedures. These factors can influence which surface features contribute to the calculated *R*_a_, particularly for the irregular surfaces produced by additive manufacturing. Although the absence of reference values prevents a direct assessment of accuracy, the comparable measurement magnitudes and consistent trends support the applicability of the proposed SAM pipeline to additively manufactured metallic surfaces.

### 3.3. Repeatability of the SAM Measurements

The repeatability of the proposed SAM method was evaluated using seven reference standards and three additively manufactured cubes. For each specimen, 10 fixed physical lines were measured 21 times under unchanged measurement conditions. The mean *R*_a_, standard deviation (σ), and coefficient of variation (CV) were calculated for each line to evaluate measurement consistency. Both Gaussian-filtering formulations were investigated to assess whether the filtering procedure influenced repeatability.

Figure 14 presents the results obtained using the cutoff-matched Gaussian formulation, which was adopted for the final surface roughness evaluation. Each point represents the mean *R*_a_ of 21 repeated measurements at a fixed line, with error bars indicating ±2σ.

The repeatability results for all specimens are summarized in Table 4. Both Gaussian formulations demonstrated low measurement variability across the investigated roughness range. For the cutoff-matched formulation, the mean CV ranged from 0.33% to 0.61% for all specimens except the 0.8μm standard, which showed a higher value of 1.91%. This higher relative variation is mainly associated with its small mean *R*_a_, since the CV expresses the standard deviation relative to the measured mean. Nevertheless, the mean within-line standard deviation for this specimen was only 0.009μm, indicating low absolute variability even at the smallest investigated roughness level.

Although the two Gaussian formulations produced different mean *R*_a_ values, their coefficients of variation remained similarly low across the investigated specimens. This indicates that the choice of filtering formulation affects the calculated roughness magnitude but has a limited influence on measurement repeatability. Overall, these findings demonstrate that the proposed SAM pipeline provides consistent roughness measurements under repeated acquisition conditions for both reference standards and additively manufactured metallic surfaces.

## 4. Discussion

The findings of this study demonstrate the potential of SAM for quantitative surface roughness evaluation of additively manufactured metallic components. By identifying the surface echo in B-scan images and converting its position into a calibrated surface profile, the proposed pipeline was able to distinguish different roughness levels across seven reference standards, ranging from 0.8 to 50μm. The comparison with established optical instruments further supported this capability. For the three standards measured using all three methods, SAM achieved a mean absolute deviation of 0.75μm from the nominal values, compared with 1.30μm for the VHX-7000 and 2.85μm for the VK-X3050. Nevertheless, the deviations in the measurements did not follow a consistent trend with increasing roughness, suggesting that measurement accuracy depends not only on the roughness magnitude but also on the surface characteristics and the ability of the acoustic system to resolve them. Factors such as surface scattering, echo localization, and the finite acoustic focal spot may contribute to these differences.

Beyond surface acquisition, the processing of the extracted profiles was found to play an important role in the calculated roughness values. The cutoff-matched Gaussian formulation consistently produced lower *R*_a_ values than the simplified approach, improving agreement with the nominal values at 25 and 50μm, while increasing the deviations at lower roughness levels. This demonstrates that the influence of filtering depends on the surface characteristics and the selected S- and L-filter nesting indices. The comparison therefore highlights the importance of clearly defined Gaussian filtering parameters for reliable surface roughness evaluation.

The reference-based evaluation provided a basis for comparing SAM with established optical measurement techniques on the additively manufactured cubes. Since the cubes had no known reference roughness values, the comparison focused on agreement between the different instruments. SAM produced *R*_a_ values comparable to those obtained using the optical instruments and showed closer agreement with the VHX-7000, with a mean absolute difference of 0.74μm. The remaining differences may be attributed to the complex surface features of additively manufactured parts, which can be captured differently depending on the measurement principle, spatial resolution, and evaluated surface region.

The repeatability results further demonstrate the stability of the proposed SAM pipeline under unchanged measurement conditions. Both Gaussian formulations showed consistently low measurement variability, indicating that their influence on the calculated *R*_a_ values does not substantially affect repeatability. Although the 0.8μm standard exhibited a higher coefficient of variation, its small absolute standard deviation suggests that this is mainly related to the low measured roughness rather than substantial measurement fluctuations. Importantly, the consistency of repeated measurements at fixed positions should be distinguished from measurement accuracy and the natural roughness variations across different surface locations. These results demonstrate that SAM provides consistent measurements at fixed positions, while evaluating multiple surface profiles is necessary to account for spatial variations in surface roughness.

Taken together, these findings demonstrate the potential of SAM as a non-contact technique for quantitative surface roughness evaluation, with the additional capability of supporting subsurface and interfacial inspection. The proposed pipeline achieved measurement performance comparable to established optical techniques while maintaining high repeatability across the investigated specimens. Nevertheless, the study was limited to a single 125MHz transducer, mainly planar surfaces, and reference-based validation of *R*_a_. Furthermore, the fixed lateral sampling interval of 2.0μm may limit the detection of finer surface features, particularly at lower roughness levels. Despite these limitations, the results provide a strong basis for the application of SAM to surface roughness assessment of additively manufactured metallic components.

## 5. Conclusions

This study established a complete procedure for extracting surface profiles from SAM B-scans and calculating quantitative roughness parameters. Evaluation using seven reference standards showed that the method could distinguish different roughness levels, while repeated measurements demonstrated high precision at fixed positions. The area-based sampling strategy also accounted for spatial surface variations and provided a more representative assessment than a single profile. SAM should therefore be considered a promising complementary technique that can combine non-contact roughness evaluation with other acoustic inspection tasks.

The next stage should extend this study toward a more comprehensive metrological validation. This will require traceably calibrated standards covering a wider range of roughness values, additional materials and surface types, and co-located measurements using SAM and established reference instruments. Future work should also evaluate the feasibility of using SAM to characterize the roughness of internal surfaces and complex triply periodic minimal surface (TPMS) lattice structures, where access using conventional measurement methods is limited. However, such geometries introduce challenges related to acoustic access, surface inclination, focusing, and multiple reflections, which may affect the reliability of surface echo detection. The effects of transducer frequency, focusing, gain, water temperature, and filtering parameters should also be evaluated systematically to determine their influence on the calculated roughness. Finally, repeated measurements after specimen repositioning and under different operators and measurement days are needed to assess reproducibility and establish a complete measurement uncertainty. These investigations will define the operating limits of the method and determine its suitability for reliable surface roughness measurement in research and industrial applications.

## Figures and Tables

**Figure 1:**
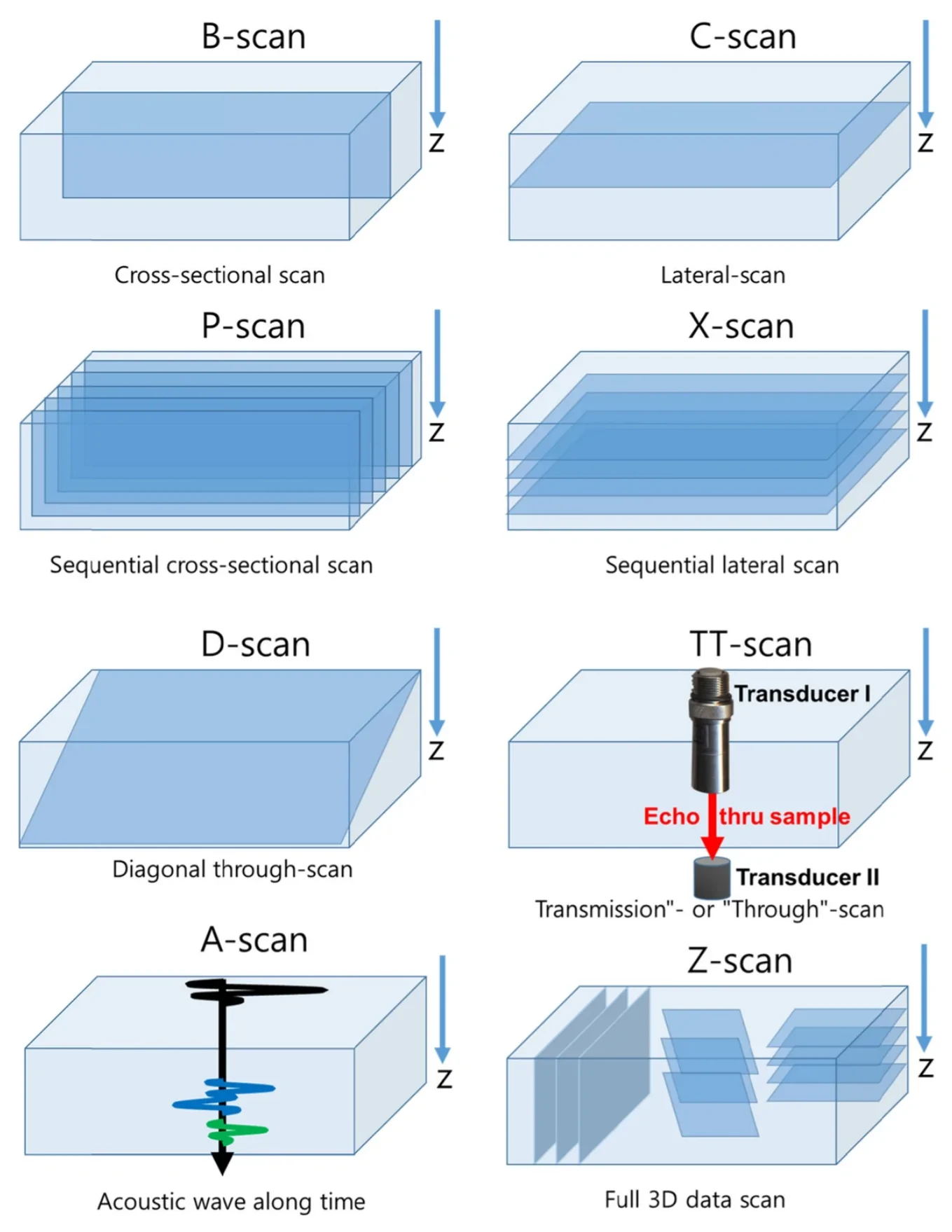
Schematic representation of the different SAM scan modes [10]. Shaded planes indicate the scan sections or imaging planes; blue arrows show the *z*-direction. In the TT-scan, the red arrow indicates the acoustic wave transmitted through the sample from Transducer I to Transducer II.

**Figure 2:**
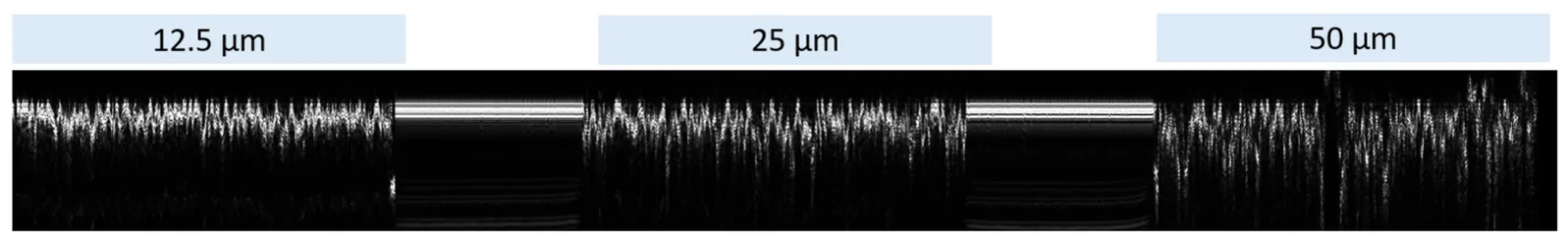
Representative SAM B-scan acquired along a path crossing the three regions of the MICROSURF 334 reference plate (SMT-MESSZEUGE Steffen Schiller, Aschaffenburg, Germany) with nominal *R*_a_ values of 12.5, 25, and 50μm. The surface echo shows progressively greater vertical variation and irregularity as the nominal surface roughness increases.

**Figure 3:**
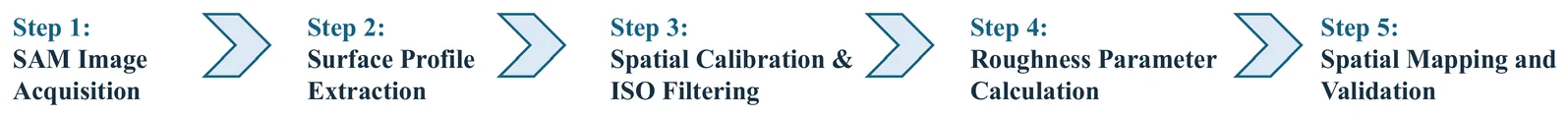
Overview of the proposed pipeline for extracting and evaluating surface roughness from SAM B-scan images.

**Figure 4:**
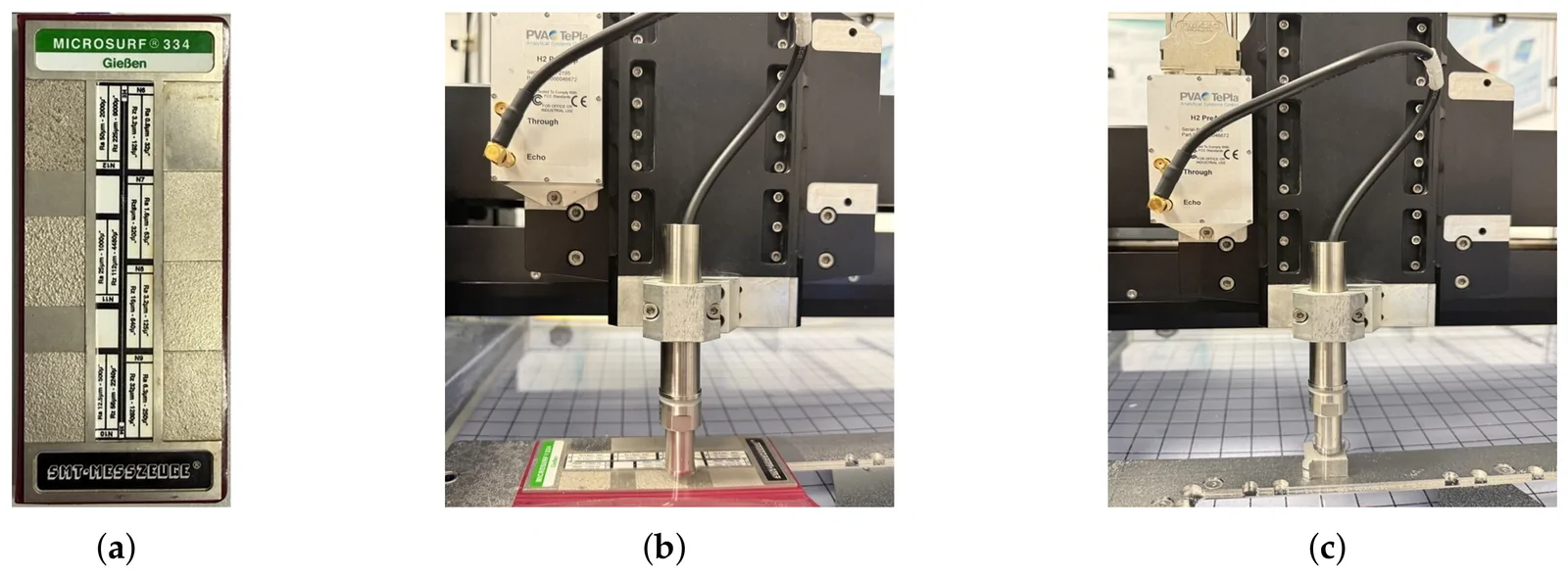
Samples and measurement arrangements used for SAM image acquisition: (**a**) MICROSURF 334 reference plate; (**b**) SAM image acquisition from the standards specimen; and (**c**) SAM image acquisition from an additively manufactured cubic specimen.

**Figure 5:**
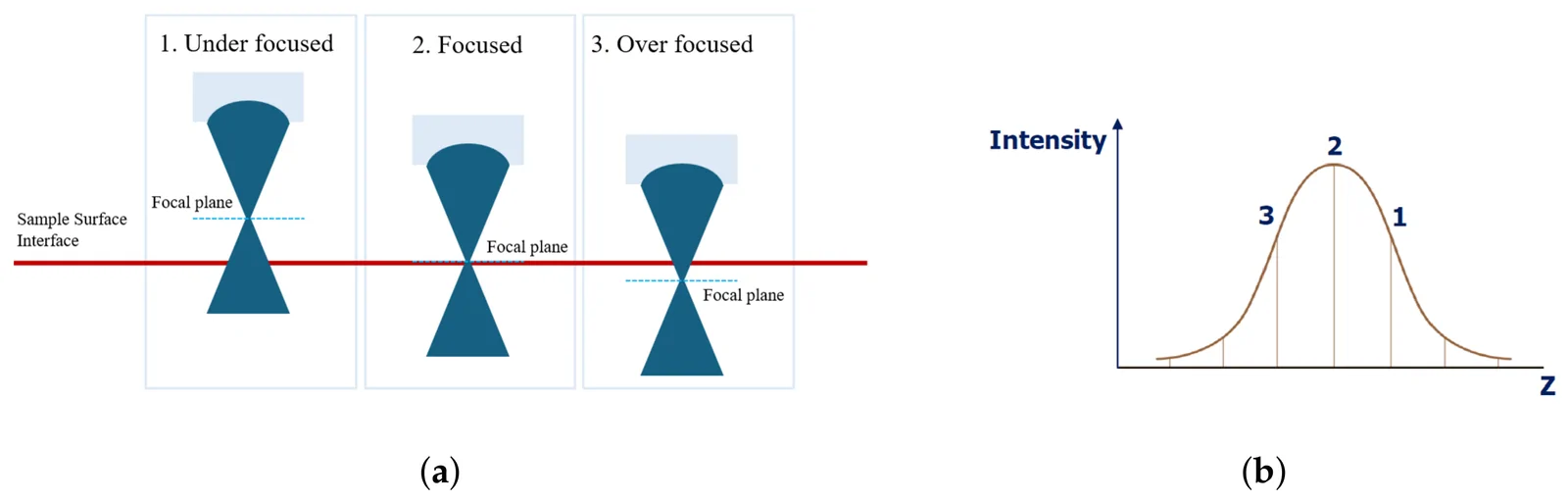
Influence of the transducer position on acoustic focusing and the reflected signal: (**a**) under-focused, focused, and over-focused positions of the specimen surface relative to the focal plane; and (**b**) variation in the reflected signal intensity with the axial (z) transducer distance to sample, where the maximum intensity is obtained at the focal position (2).

**Figure 6:**
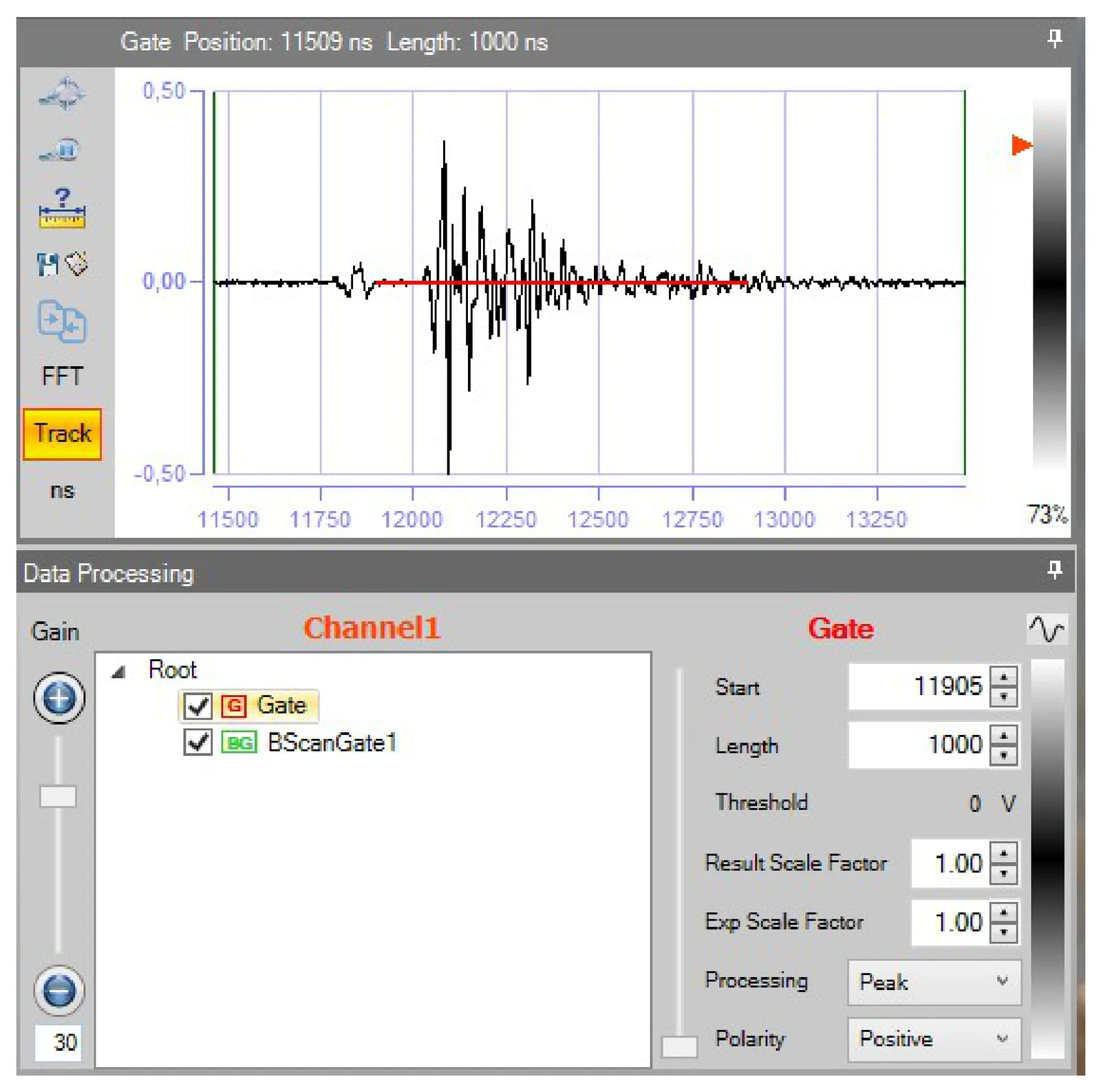
WinSAM 8 interface showing the main acquisition settings and the percentage scale used to monitor the gated surface echo amplitude during adjustment of the transducer focus and the receiver gain. The displayed time axis spans 11,500–13,500 ns; the gate starts at 11,509 ns and has a length of 1000 ns. The vertical scale ranges from −0.50 to 0.50, and the percentage indicator shows 73%.

**Figure 7:**
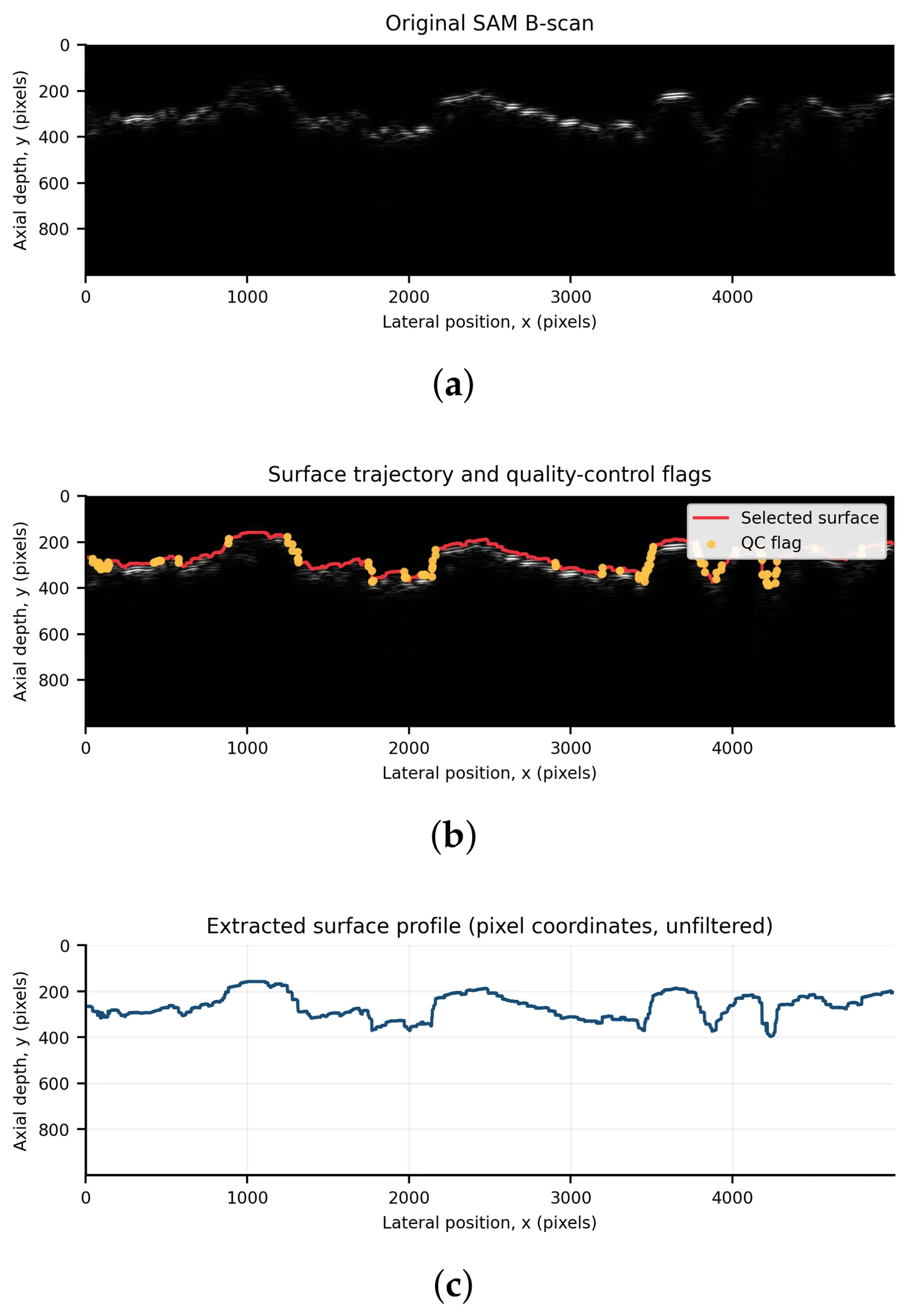
Surface-profile extraction from a representative SAM B-scan: (**a**) original grayscale B-scan image; (**b**) detected surface trajectory with quality control indicators, where the yellow dots highlight potentially uncertain surface positions requiring further inspection; and (**c**) final extracted surface profile in pixel coordinates, before spatial calibration and subsequent profile filtering.

**Figure 8:**
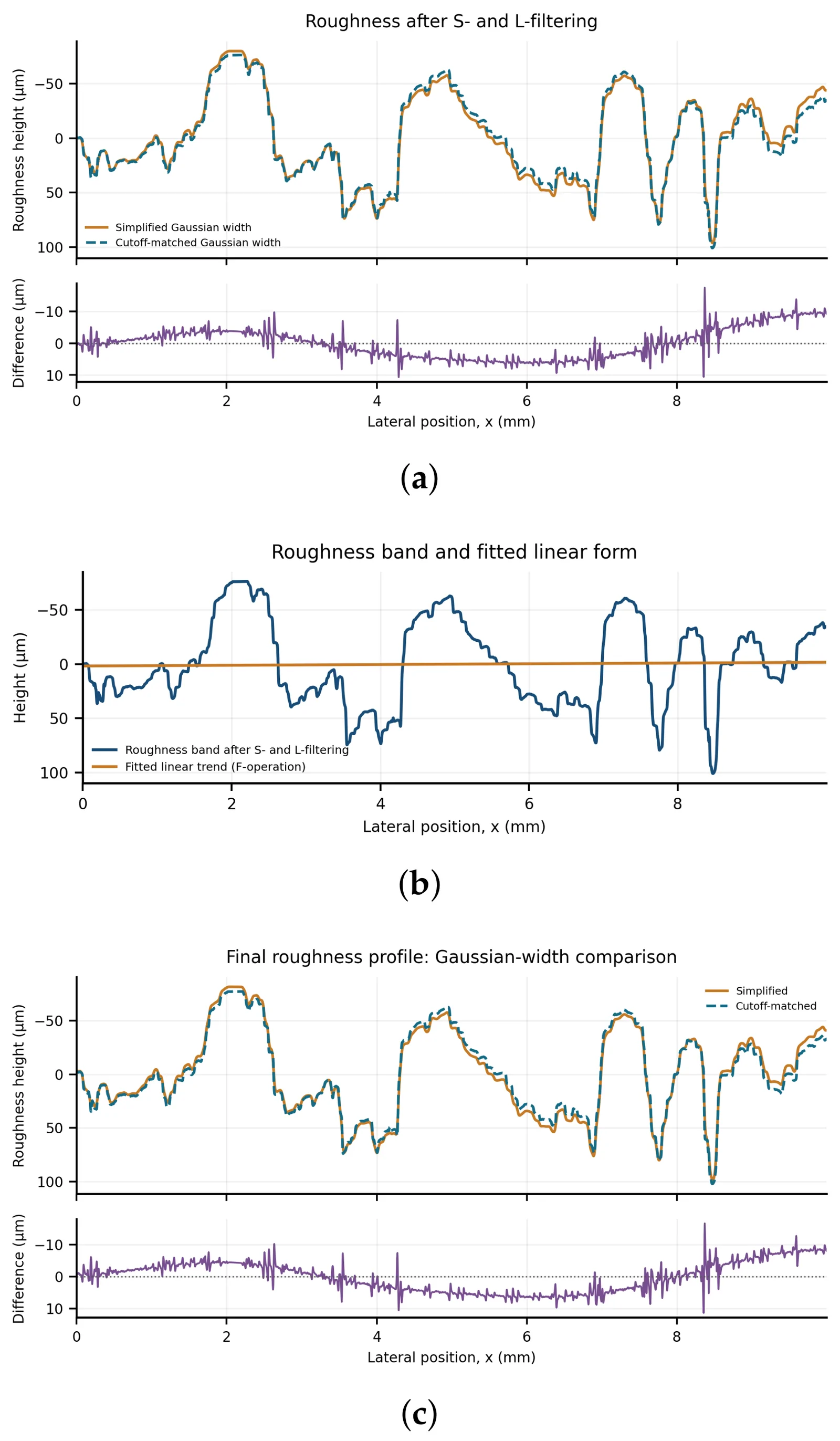
Comparison of surface-profile filtering using simplified and cutoff-matched Gaussian formulations: (**a**) roughness profiles after S- and L-filtering and their difference; (**b**) roughness-band profile and fitted linear trend; and (**c**) final roughness profiles after form removal and their difference.

**Figure 9:**
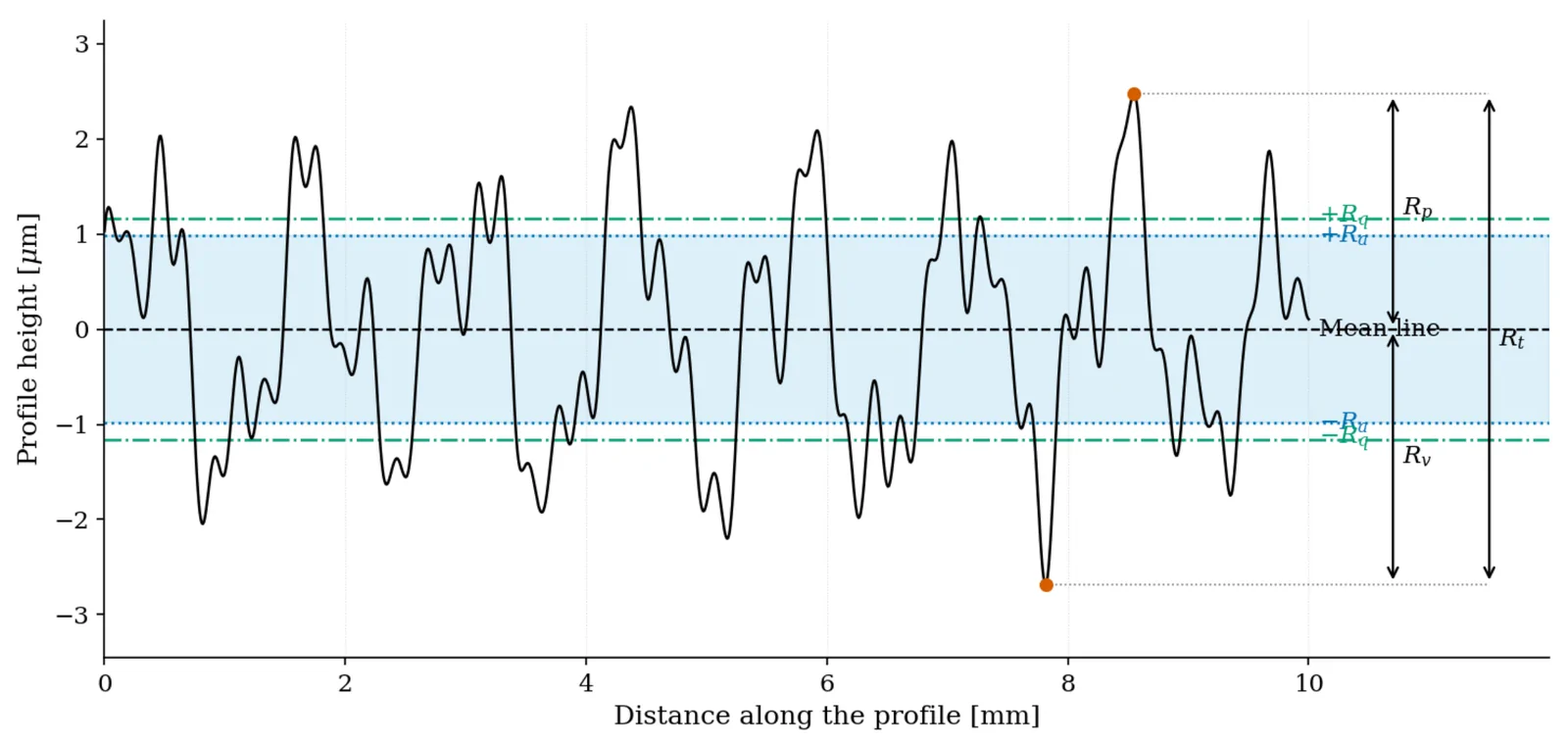
Schematic representation of the profile roughness parameters *R*_a_, *R*_q_, *R*_p_, *R*_v_, and *R*_t_.

**Figure 10:**
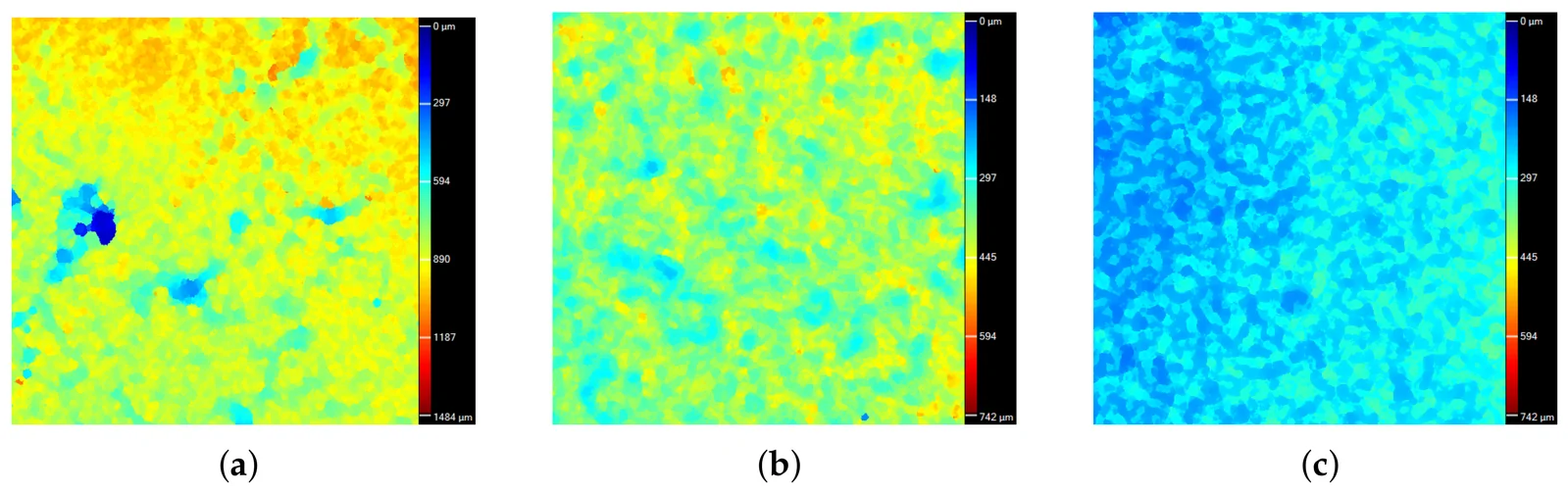
Representative SAM C-scan images of surface roughness reference standards with nominal *R*_a_ values of (**a**) 50μm, (**b**) 25μm, and (**c**) 12.5μm, illustrating spatial variations in the acoustic response across their surfaces.

**Figure 11:**
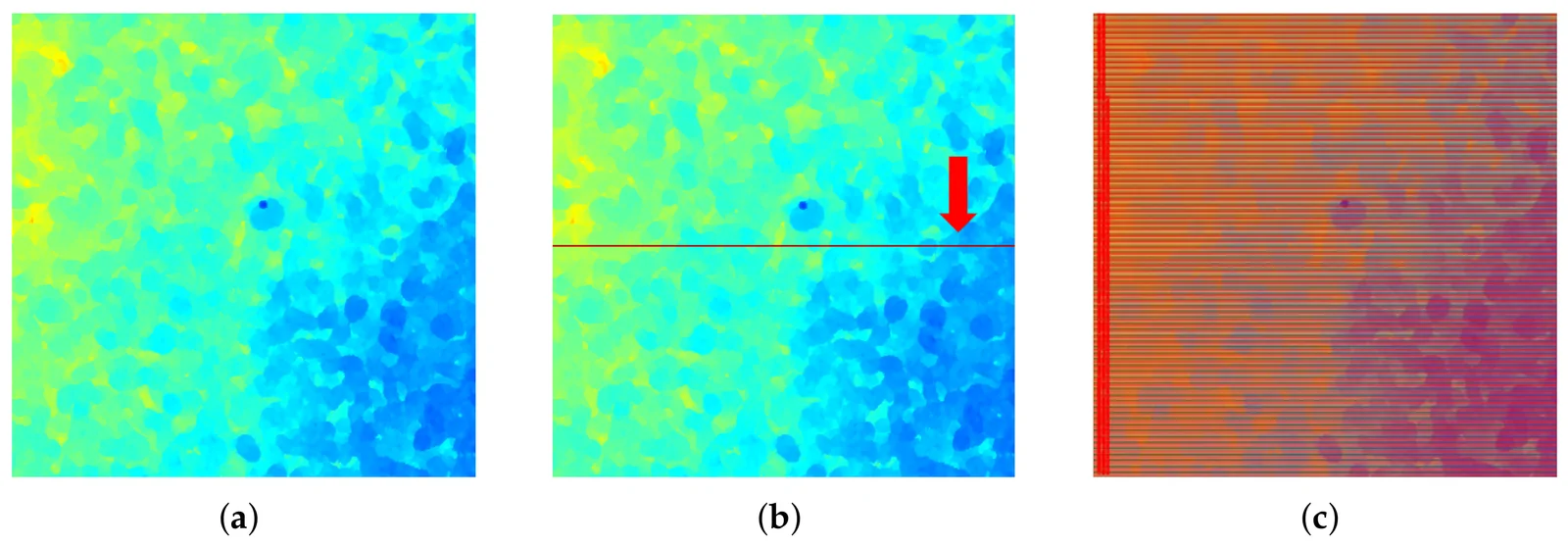
Spatial sampling strategy used for the area-based surface roughness evaluation: (**a**) SAM C-scan of the complete 10mm×10mm measurement area; (**b**) location of one selected B-scan measurement line; and (**c**) positions of the 500 parallel B-scans acquired at a spatial offset of 0.02mm.

**Figure 12:**
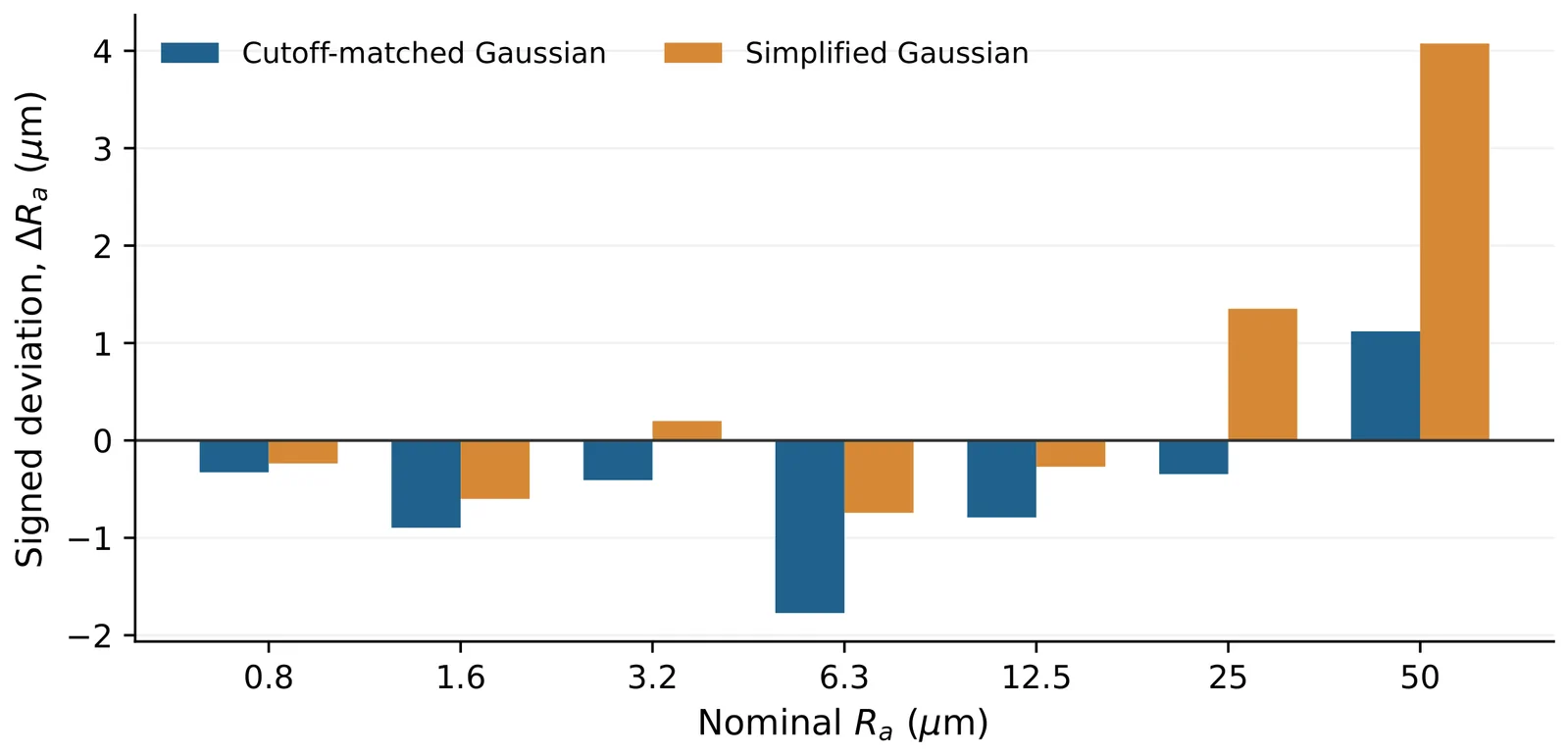
Signed deviations of SAM-measured *R*_a_ values from the nominal roughness values of the seven reference standards, using simplified and cutoff-matched Gaussian filtering.

**Figure 13:**
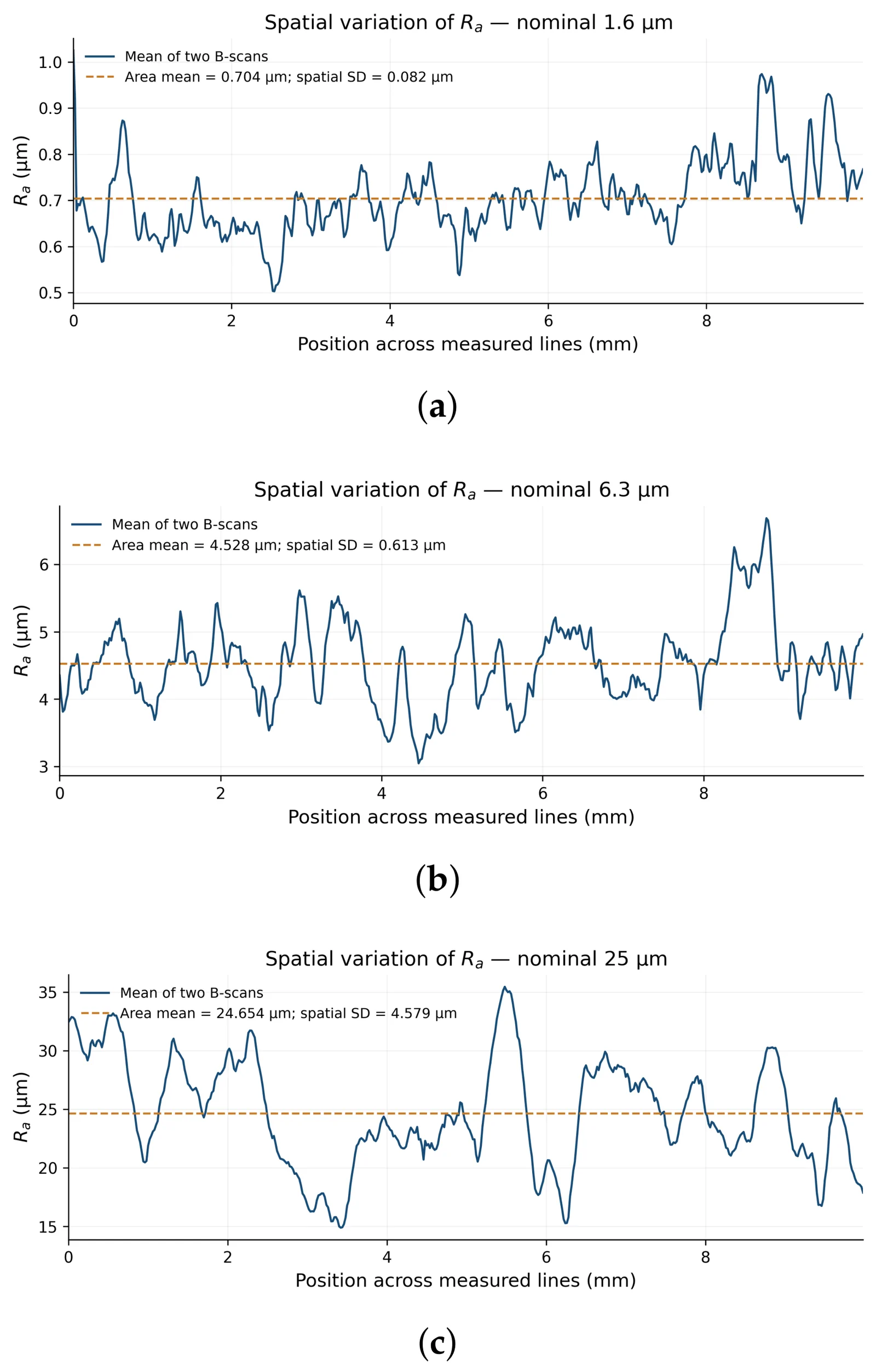
Spatial variation of SAM-measured *R*_a_ across 500 physical line positions for reference standards with nominal roughness values of (**a**) 1.6μm, (**b**) 6.3μm, and (**c**) 25μm. Each plotted value represents the mean *R*_a_ obtained from two repeated B-scans acquired at the corresponding physical line position.

**Figure 14:**
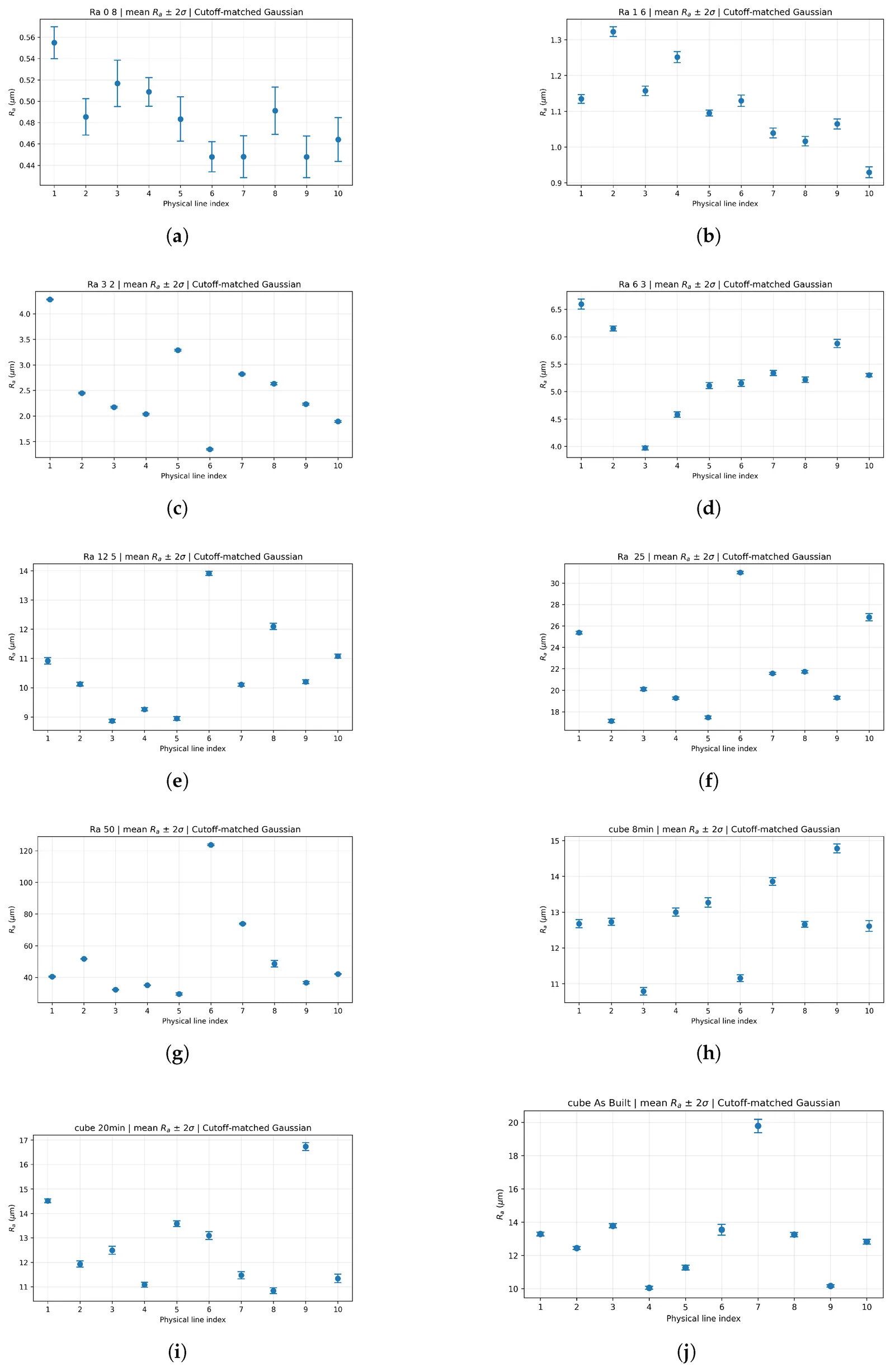
Repeatability of SAM-based *R*_a_ measurements using the cutoff-matched Gaussian formulation for reference standards with nominal values of (**a**) 0.8, (**b**) 1.6, (**c**) 3.2, (**d**) 6.3, (**e**) 12.5, (**f**) 25, and (**g**) 50μm, and the additively manufactured specimens (**h**) Cube 8 min, (**i**) Cube 20 min, and (**j**) Cube as-built.

**Table 1:** Profile-filter nesting indices for the surface roughness ranges investigated in this study, based on ISO 21920-3 [19].

*R*_a_ Upper Tolerance *U*	L-Filter Nesting Index $N_{\mathrm{ic}}$	S-Filter Nesting Index $N_{\mathrm{is}}$
(μm)	(μm)	(μm)
0.1<U≤2	800	2.5
2<U≤10	2500	8
U>10	8000	25

**Table 2:** Comparison of measured *R*_a_ values and their absolute deviations from the nominal values of the seven reference standards.

Nominal *R*_a_	Method	Measured *R*_a_	Absolute Deviation
(μm)		(μm)	(μm)
0.8	SAM (cutoff-matched)	0.473	0.327
0.8	SAM (simplified)	0.564	0.236
1.6	SAM (cutoff-matched)	0.704	0.896
1.6	SAM (simplified)	1.000	0.600
3.2	SAM (cutoff-matched)	2.792	0.408
3.2	SAM (simplified)	3.399	0.199
6.3	SAM (cutoff-matched)	4.528	1.772
6.3	SAM (simplified)	5.557	0.743
12.5	SAM (cutoff-matched)	11.710	0.790
12.5	SAM (simplified)	12.230	0.270
12.5	VHX-7000	11.570	0.930
12.5	VK-X3050	11.910	0.590
25	SAM (cutoff-matched)	24.654	0.346
25	SAM (simplified)	26.352	1.352
25	VHX-7000	26.490	1.490
25	VK-X3050	23.600	1.400
50	SAM (cutoff-matched)	51.120	1.120
50	SAM (simplified)	54.075	4.075
50	VHX-7000	51.480	1.480
50	VK-X3050	56.550	6.550

**Table 3:** Comparison of the *R*_a_ values measured on the additively manufactured cubic specimens.

Specimen	SAM *R*_a_	VHX-7000 *R*_a_	VK-X3050 *R*_a_
	(μm)	(μm)	(μm)
Cube 1 (as-built)	10.984	11.21	14.74
Cube 2 (8 min)	12.428	11.81	14.87
Cube 3 (20 min)	12.737	11.37	15.37

**Table 4:** Summary of SAM repeatability results for the reference standards and additively manufactured cubes using both Gaussian-filtering formulations. For each specimen, 10 fixed physical lines were measured 21 times. The standard deviation (SD) and coefficient of variation (CV) were calculated separately from the 21 repeated measurements at each line. The reported mean SD and mean CV represent the averages of these values across the 10 physical lines.

Specimen	Gaussian Method	Mean of Line Means	Mean SD Within Each Line	Mean CV
		(μm)	(μm)	(%)
*R*_a_ 0.8	Cutoff-matched	0.485	0.009	1.91
	Simplified	0.586	0.010	1.70
*R*_a_ 1.6	Cutoff-matched	1.114	0.007	0.61
	Simplified	1.451	0.008	0.56
*R*_a_ 3.2	Cutoff-matched	2.515	0.007	0.33
	Simplified	3.319	0.011	0.35
*R*_a_ 6.3	Cutoff-matched	5.329	0.027	0.51
	Simplified	6.730	0.025	0.37
*R*_a_ 12.5	Cutoff-matched	10.546	0.036	0.34
	Simplified	10.928	0.039	0.36
*R*_a_ 25	Cutoff-matched	21.975	0.073	0.34
	Simplified	23.394	0.078	0.34
*R*_a_ 50	Cutoff-matched	51.362	0.265	0.59
	Simplified	54.648	0.284	0.60
Cube 8 min	Cutoff-matched	12.752	0.056	0.44
	Simplified	13.022	0.056	0.44
Cube 20 min	Cutoff-matched	12.707	0.068	0.54
	Simplified	12.754	0.069	0.55
Cube as-built	Cutoff-matched	13.038	0.081	0.59
	Simplified	13.614	0.090	0.64

## Data Availability

The original contributions presented in this study are included in the article. Further inquiries can be directed to the corresponding author.
